# What is Interpretability?

**DOI:** 10.1007/s13347-020-00435-2

**Published:** 2020-11-12

**Authors:** Adrian Erasmus, Tyler D. P. Brunet, Eyal Fisher

**Affiliations:** 1 Institute for the Future of Knowledge, University of Johannesburg, Johannesburg, South Africa; 2 Department of History and Philosophy of Science, University of Cambridge, Free School Ln., Cambridge, CB2 3RH UK; 3 Cancer Research UK Cambridge Institute, University of Cambridge, Li Ka Shing Centre, Robinson Way, Cambridge, CB2 0RE UK

**Keywords:** Interpretability, Explainability, XAI, Medical AI

## Abstract

We argue that artificial networks are explainable and offer a novel theory of interpretability. Two sets of conceptual questions are prominent in theoretical engagements with artificial neural networks, especially in the context of medical artificial intelligence: (1) Are networks *explainable*, and if so, what does it mean to explain the output of a network? And (2) what does it mean for a network to be *interpretable*? We argue that accounts of “explanation” tailored specifically to neural networks have ineffectively reinvented the wheel. In response to (1), we show how four familiar accounts of explanation apply to neural networks as they would to any scientific phenomenon. We diagnose the confusion about explaining neural networks within the machine learning literature as an equivocation on “explainability,” “understandability” and “interpretability.” To remedy this, we distinguish between these notions, and answer (2) by offering a theory and typology of interpretation in machine learning. Interpretation is something one does to an explanation with the aim of producing another, more understandable, explanation. As with explanation, there are various concepts and methods involved in interpretation: *Total* or *Partial*, *Global* or *Local*, and *Approximative* or *Isomorphic*. Our account of “interpretability” is consistent with uses in the machine learning literature, in keeping with the philosophy of explanation and understanding, and pays special attention to medical artificial intelligence systems.

## Introduction

Two sets of conceptual problems have gained prominence in theoretical engagements with artificial neural networks (ANNs). The first is whether ANNs are *explainable*, and, if they are, what it means to explain their outputs. The second is what it means for an ANN to be *interpretable*. In this paper, we argue that ANNs are, in one sense, already explainable and propose a novel theory of interpretability.

These issues often arise in discussions of medical AI systems (MAIS), where reliance on artificial decision making in medical contexts could have serious consequences. There is evidence that some of these systems have superior diagnostic and predictive capabilities when compared to human experts (Esteva et al. 2017; Fleming 2018; Rajpurkar et al. 2017; Tschandl et al. 2019). Indeed, many MAIS are already deployed in the clinic, including algorithms aimed at diagnosing retinal disease (De Fauw et al. 2018), and breast cancer treatment recommendations (Somashekhar et al. 2018) and screening (McKinney et al. 2020). For some, accomplishments like these are a precursor to the promising incorporation of machine learning (ML) into effective medical decision making (Wiens and Shenoy 2018). However, others have noted problems facing MAIS, including vulnerabilities to nefariously motivated adversarial attacks by various stakeholders in the healthcare system (Finlayson et al. 2019) and algorithmic racial biases in the prediction of future health care needs due to objective functions and training procedures (Obermeyer et al. 2019).

These problems have led to calls for MAIS, and ANNs in general, to be explainable—that is, if an ANN makes a recommendation, there should be an explanation for its decision (Athey 2017; Aler Tubella et al. 2019). In the context of healthcare, motivated by the need to justify artificial decisions to patients, some argue that maximizing the benefits of MAIS requires that their outputs be explainable (Watson et al. 2019). Others argue that having explainable MAIS makes it easier to recognize and remedy algorithmic rules which lead to inaccurate outputs (Caruana et al. 2015). On this view, having an explanation allows us to evaluate a MAIS’ recommendations, which some argue is necessary for assessing the trustworthiness of such systems (see Ribeiro et al. 2016; Mittelstadt et al.^2019^).

Issues of explainability have become problematic due to the prominent view that the accuracy of an AI system trades off against its explainability. We refer to this as the Accuracy-Explainability (*AE*) trade-off (Gunning 2017; London 2019). Many of the problems MAIS are designed to solve are complicated enough that achieving a high degree of accuracy requires highly complex ANNs. This, conjoined with the assumption that highly complex ANNs are “less explainable,” leads to the peculiar reasoning that if an AI system being more accurate entails low explainability, and if low explainability entails an inability to assess trustworthiness, then a highly accurate AI system entails an inability to assess trustworthiness. However, this strikes us as unintuitive since, presumably, accuracy should count towards trustworthiness.

There are three common strategies for dealing with this problem. Some argue that simplicity should be favored over accuracy when developing MAIS (Adkins 2017; Athey 2017). Others argue that we simply need to develop ways of increasing explainability (Gunning 2017). And for some, there is no problem at all since either we face similar issues when dealing with human decision makers (London 2019; Zerilli et al. 2019) or because simpler models may sometimes achieve the same degree of accuracy as more complex algorithms for the same task (Rudin 2019). Yet, all these approaches turn on what we mean by *explanation* and what features make explanations “good” or “fitting” for a given account. The literature on ANNs in general, and MAIS in particular, often makes use of the concepts of *explainability*, *understandability* and *interpretability*, but offer little critical engagement and contain persistent disagreement on the definitions of these terms (Lipton 2018; Krishnan 2019). Moreover, a central problem plaguing the ML literature is the conflation of these concepts: Explainability and understandability are treated as if they are synonymous with interpretability.

Several philosophers have analyzed these concepts more carefully. Zednik (2019), for instance, offers a pragmatic account of *opacity* to provide a normative framework detailing different kinds of knowledge that ought to be required by different stakeholders. Explanations, on this view, are the basis for how such knowledge is acquired. Creel’s (2020) account of *transparency* aims at illuminating recent successes in reducing algorithmic opacity and providing AI-explanations. Krishnan (2019) argues that pursuing definitions of interpretability, and related terms like explainability and understandability, is misguided because doing so reduces solutions to opacity-related problems to merely finding ways to make ANNs more transparent. Her claim that there are other solutions to these problems, while compelling, does not entail that defining interpretability in the context of ANNs should be wholly abandoned, particularly given the apparent conflation of terms. Páez (2019) recommends that focus should be shifted from explanation to understanding, arguing that traditional explanations of opaque systems are impossible and that understanding can be acquired by other means which do not require such explanations. Such strategies, Páez argues, exist in ML in the form of interpretive models aimed at understanding how an ANN functions, and post hoc interpretability aimed at understanding individual decisions. Taken together these accounts go some way to establishing interpretability as an important concept in its own right and its use within ML. However, none provide an explicit account of the term (see Section 4.1) or how it should be used in analyses of ANNs (Sections 4.2–4.4).

Our first aim is to clearly set the concepts of explainability, understandability, and interpretability apart. The explosion of different notions of “explanation” in the context of AI has reinvented the wheel; philosophers of science have been developing notions of scientific explanation for nearly a century (Section 2). In contrast to Páez’s (2019) claim that traditional explanations of ANNs are impossible, we argue that four such accounts—the *Deductive Nomological*, *Inductive Statistical*, *Causal Mechanical*, and *New Mechanist* models—indeed apply to neural networks, as they would to any scientific phenomenon (Section 3.1). In this sense there is no AE trade-off. The source of much confusion within the literature is the conflation of “explainability,” “understandability,” and “interpretability” in cases where they are not interchangeable. Many claims within and surrounding the ML literature are explicitly lodged as calls for “explainability,” when it is the understandability of existing explanations that should be at issue. We briefly unpack the relationship between understanding and explanation, showing that it is understanding that is defeasible by increasing complexity (Section 3.2).

We then provide an explicit account of interpretability and offer a typology of interpretation methods (Section 4). We argue that interpretation is a relation between two explanations. During the process of interpretation, one explanation gives rise to a *more understandable* explanation (Section 4.1). As with explanation, there are varieties of interpretation: *Total* or *Partial* (Section 4.2), *Global* or *Local* (Section 4.3), and *Approximative* or *Isomorphic* (Section 4.4). Our account of interpretability is consistent with many uses within AI, in keeping with philosophy of explanation and understanding, and provided with special attention to the accuracy-complexity relationship in MAIS.

## The Indefeasibility of Explanation

In the aim for explainable AI (XAI) we ought to work with well-theorized conceptions of *explanation*. There are many accounts of scientific explanation; we deploy those most germane to the problem of separating out “interpretability” as a special epistemic activity. While the recent uses of concepts of “interpretability” and “interpretation” are variously and sometimes inconsistently defined (see Section 4), “explanation” has a far longer and more rigorous conceptual history. Philosophers have reflected on the nature of explanation since before Socrates, but the modern discussion began in the late 1940s with Hempel and Oppenheim (1948).

We focus on the four models of explanation that have received the most significant attention: The Deductive Nomological model; the Inductive Statistical model; the Casual Mechanical model; and, more recently, the New Mechanist model. In this section, we will briefly outline each of these models and argue that explanations of each variety are indefeasible to complexity; that is, increasing the complexity of a phenomenon does not make it any less explainable.^1^ Establishing the indefeasibility of explanation is the first step to recognizing that the alleged trade-off between accuracy (complexity) of ANNs and their explainability is misguided. Conceiving of this trade-off in terms of “explainability” has led to confusion in the debates surrounding interpretability and trust in MAIS. We argue that when there is a trade-off at all, it is with understanding, not explanation.

### Four Kinds of Explanation

**Fig. 1:**
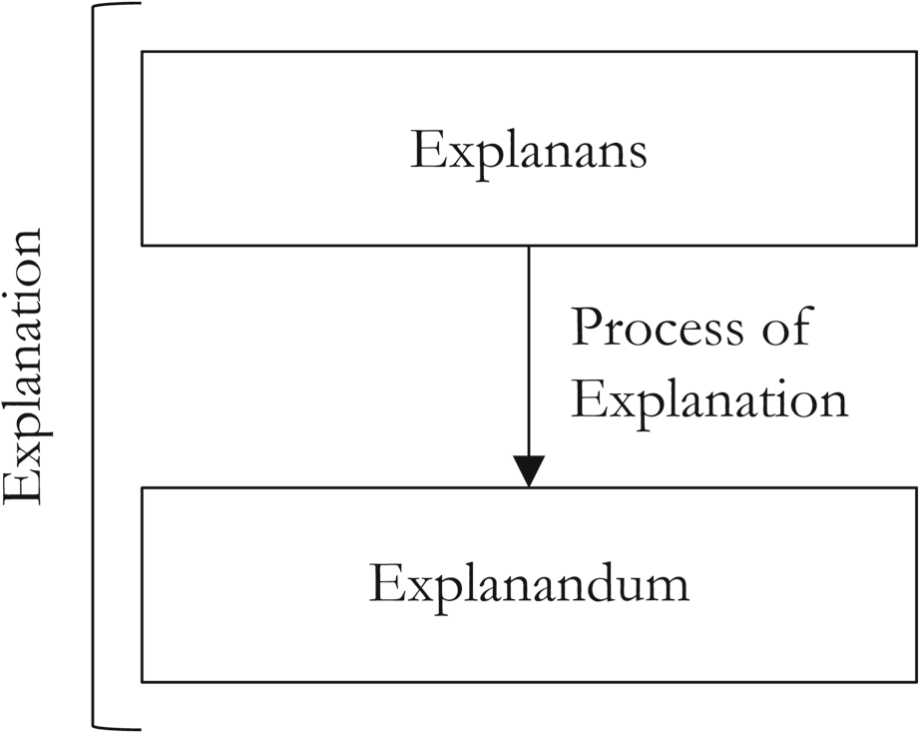
The general structure of explanation

Each model of explanation has the same broad structure. An explanation consists of an *explanandum*, an *explanans* and some *process of explanation* connecting the explanans to the explanandum (see Fig. 1).

**Fig. 2:**
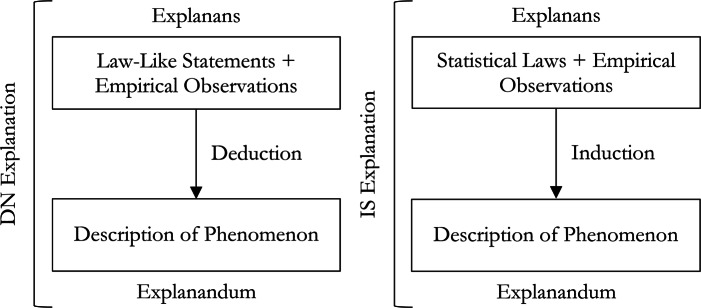
The structures of DN and IS explanation

For *Deductive Nomological* (DN) explanation (see Fig. 2) in particular, a successful explanation must satisfy two conditions (Hempel and Oppenheim 1948; Hempel 1965). First, the process of explanation must take the form of a sound *deductive* argument. Second, the explanans must have an essential *nomic* premise, i.e., at least one *law of nature* or *law-like proposition* without which the deduction would be invalid. For example, the movement of a steel bearing on a flat table can be explained using the law, “all ferrous metals are physically attracted to magnetic fields” in conjunction with the fact that magnet has been brought close to the bearing.

While the DN model is good for illustrating the explanation of phenomena which result from deterministic laws, it does not capture the characteristics of probabilistic events. In response to this, Hempel (1965) introduced *Inductive Statistical* (IS) explanation. IS explanation involves the inference of an individual event from a *statistical law* and empirical information about the event (see Fig. 2). For example, the increased probability of having breast cancer given a mutated *BRCA1* gene in conjunction with a particular patient having a mutated *BRCA1* gene explains the patient having breast cancer. Here, the relation between the explanandum and the explanans is inductive because all that can be inferred from the information given is there being a higher or lower probability that the patient has breast cancer. If the probability of having breast cancer given that the patient has a mutated *BRCA1* gene were lower, then even if the patient has breast cancer, this information cannot be used to explain it. The basic idea then is that the success of an IS explanation depends on whether the explanans entails a higher probability of the explanandum obtaining.

**Fig. 3:**
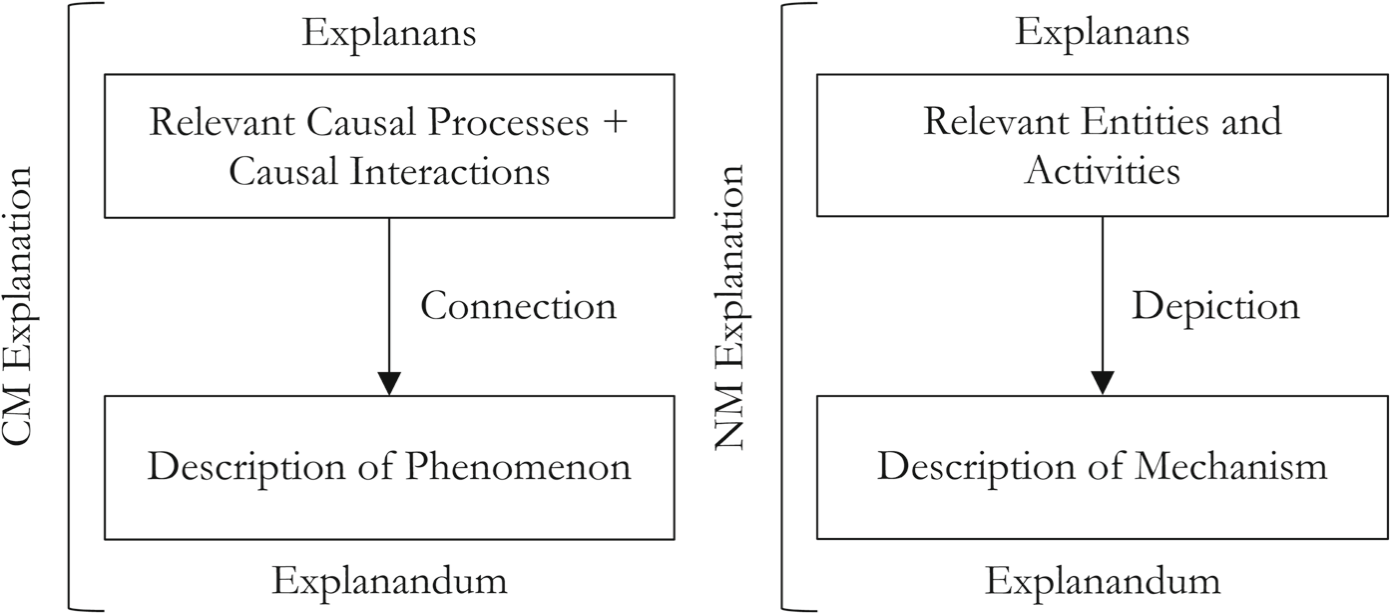
The structures of CM and NM explanation

Two decades after Hempel’s work on the DN and IS models, Salmon (1984) proposed the *Causal Mechanical* (CM) model, which he claims highlights the central role of causation in explanation.^2^ The idea behind the CM model is that explanation involves showing how the explanandum fits into the causal structure of the world. There are two important aspects of causation which feature in CM explanations. The most basic of these, a *causal process*, is the ability to transfer a *mark* or its own physical structure in a spatio-temporally continuous way. An example of this would be the movement of sound waves through air, or the movement of a particle through space. The second, a *causal interaction*, occurs when causal processes interact with one another resulting in a modified structure. Examples include sound wave interference, or the collision of particles. A successful CM explanation involves citing some parts of the causal processes and causal interactions which lead to the phenomenon in question (see Fig. 3). We might, for instance, explain the phenomenon of noise cancellation by citing the existence of two sound waves, one with inverted phase to the other (the causal processes) interfering with one another (the causal interaction).

A cluster of views of explanation has recently emerged, all termed *New Mechanist* (NM). These views all center on the idea that providing a mechanism is important for explanation, and originate largely from the work of Machamer et al. (2000), Bechtel (2011), and Craver and Darden (2013). The idea behind NM accounts is that providing an explanation involves showing how some phenomena arise from a collection of *entities* and *activities* (see Fig. 3).^3^^,^^4^ A successful explanation involves identifying the entities and activities that bring about a phenomenon with regularity and without gaps, missing entities or activities. For example, an explanation of protein synthesis will need to identify the entities involved (ribosomes, aminoacyl-tRNAs, mRNAs, GTP, etc.) and the activities (binding of mRNA to the ribosome, hydrolysis of GTP, translocation of mRNA within the ribosome, etc.), which are typically *depicted* in the form of a mechanistic diagram of the sort familiar from textbook mechanisms. A mechanism also includes two special collections of entities and activities, the initial or “set-up” and the final or “termination” conditions, in this case the binding of mRNA to the ribosome and the final release of a synthesized protein respectively. However, to better describe biological mechanisms exhibiting cyclic organizations, some argue that such conditions are not necessary for mechanistic explanations (Bechtel 2011). Yet, since ANNs are never cyclic, Machamer et al.’s characterization of mechanisms is apt for explaining such systems. Moreover, when ANNs are recurrent, the pathways can always be unrolled and mechanistically depicted.

There are several criticisms of the accounts of explanation described above (see Salmon 1989; Skillings 2015; Godfrey-Smith 2016). While we do not have the space to engage with all of these, there are two that may have implications for the arguments that follow, and are thus worth addressing. First, it may be argued that some of the typically cited challenges facing DN, IS, and CM explanations, such as explanatory asymmetry and explanatory irrelevance, are the result of disregard for, or inadequate treatment of the role of causation in explanation (Woodward 2003). Woodward’s (2003) causal-interventionist account of explanation, according to which successful explanations exhibit patterns of counterfactual dependence, may help deal with these issues, particularly when it comes to the problem of explanatory relevance. Since we argue in Section 3 that ANNs are explainable using the traditional models described above, it may be argued that our account of interpretability could inherit some of these issues. We agree that the causal-interventionist account may be useful for solving some questions about explainability, understandability, and interpretability in AI (Páez 2019). Indeed, we describe interpretability processes in Section 4.3 some of which result in understandable explanations which include counterfactual information, however, the causal-interventionist account includes pragmatic elements that we maintain should not count against a phenomenon’s *explainability*, namely that an explanation should be “deeper or more satisfying” (Woodward 2003: 190) than those provided by DN, IS, or CM explanations. Of course, we agree that some explanations can be more preferable than others given some explanatory virtue, such as simplicity, but we disagree that such virtues should be considered when evaluating the *explainability* of a phenomenon.

Second, proponents of the ontic conception of explanation, according to which explanations are physical composites of the entities being represented, may argue that the characterization of NM explanation above is misguided. Explanations, on this view, are not *depictions* or *representations* of the mechanisms which constitute the phenomenon, but rather physical entities themselves which actively contribute to the causal structure of the world. Accordingly, there is “no question of explanations being ‘right’ or ‘wrong,’ or ‘good’ or ‘bad.’ They just are.” (Craver 2007: 27). The ontic conception supports our position regarding the indefeasability of explanation below. It does so, however, in a way we find problematic. On a strongly ontic conception of explanation, there *is* an objective explanation of ANNs, provided only that there *are* ANNs. Since there are ANNs, they would be automatically objectively explainable on this conception of NM. But this purchases the indefeasibility thesis at the cost of relevance to issues of explainability in ML. Calls for XAI are not, to our minds, calls for objective explanations.

Like Craver (2007), we hold that what makes something an explanation is not some set of pragmatic conditions such as whether it produces understanding, or whether it is a “good” explanation because it satisfies some set of explanatory virtues, but rather whether it accurately maps onto the world via any one of the explanation processes outlined above. Our concern with explanations and use of the concept of explanation are with “explanatory texts” (Craver 2007), that is, how explanations are given by scientists, ML researchers in particular. Nonetheless, our rejection of the involvement of pragmatic conditions does not entail or require the strong ontic thesis above. We only emphasize that, even if one finds an explanation undesirable because it does not satisfy some particular set of explanatory virtues or pragmatic conditions, that does not make it any less an explanation. And if there is an explanation for a given phenomenon, then that phenomenon is explainable.

### The Indefeasability Thesis

Unfortunately, the well-developed typology of explanation models described above is largely neglected in discussions of explainability in neural networks. Those who argue for the inverse relationship between complexity and explainability for ANNs have overlooked an important feature of explanation as it is understood above: its *indefeasibility*. They often treat complex MAIS as though they had outgrown their explainability, but this is not quite right. In fact, the explainability of a phenomenon—such as the output of an AI system—depends only on the relationships between our background theories and evidence, and the procedures of the explanation process used. In other words, in each of the accounts of explanation introduced above, the features that make something an explanation turn out to be invariant with respect to the complexity of both the explanans and the explanandum. What the considerations in this subsection illustrate is the following indefeasibility thesis for explanation of the phenomena characteristic of ANNs: Adding complexity to the explanation of the phenomenon does not entail that the phenomenon is any less *explainable*.

This is not a claim about the quality, superiority, or goodness of a given explanation. Our concern is whether increasing the complexity of a given explanation makes it no longer an explanation. We argue that, while increasing complexity may reduce the quality of the explanation, by making it less preferable or understandable (see Section 3.2), or even in some cases make it a bad explanation, it does *not* make it any less *an explanation*, and thus the explainability of the phenomenon of interest is unaffected. We clarify this position by looking at each kind of explanation in turn.

Consider a DN explanation of some phenomenon beginning with a simple explanans, a set of law-like premises **L** and empirical observations **E**, that concludes with the explanandum/description of a phenomenon *x* after a valid deduction. What makes this a DN explanation is just that the explanandum is reached deductively using an explanans that is partially law-like. Now consider what happens if we expand the explanans to include more premises, which are consistent with our best background theories and evidence, say by substituting one of the law-like premises for a set of *n* laws jointly entailing it. This may make the explanans less manageable or more difficult to understand, but we still have a DN explanation. That is, if *x* follows deductively from **L** + **E**, then it also follows from $\mathbf {L}^{*} = \{\mathbf {L}_{\mathbf {1}} + \mathbf {L}_{\mathbf {2}} + ... + \mathbf {L}_{\mathbf {n}}\} + \mathbf {E}$, where **L**_**1**...**n**_ are (complexity-increasing) laws which, taken together, entail **L**. The expansion of the explanans does not make its laws any less law-like nor its conclusion any less deductively valid. Put another way, the explanandum is no less explainable, since the connection to the explanans, no matter complexity, is still deduction.

We might also increase the complexity of a DN explanation by substituting the empirical observations with a more complex set of observations, say by describing them at a more concrete level of abstraction, or with more accuracy. To account for how these observations play a role in explaining the phenomenon of interest, we may require a more complex set of laws. Suppose we have the explanans **L** + **E** as before, and consider some more complex set of true laws L* and/or more complex set of empirical observations E* from which *x* follows. Since L* is a set of laws (or at least law-like propositions), and E* is a set of empirical observations, the deduction of *x* from this (more complex) pair constitutes a DN explanation of *x*. As above, the explainability of *x* does not depend on the complexity of its explanans, but on whether the relationship between explanans and explanandum is deductive. To illustrate, consider the example of the steel bearing moving on the flat table from before. We might increase the complexity of the explanation by noting more empirical observations, such as the initial distance of the magnet from the bearing, surface friction forces, and so on. Or we may explain the movement of the steel bearing by using more detailed laws including those governing the total induced magnitude of force on the ferromagnetic material’s volume, and laws of motion, among others. These laws, taken together, entail that ferrous metals are attracted to magnetic fields. While this more complex explanation may be more difficult to understand, and is by most accounts less appealing than the simpler one, it is still a DN explanation for the movement of the steel bearing on the flat table.

Importantly, not all ways of *changing* an explanans are only additions of complexity, some changes affect the quality of the explanation produced. For example, we might change a satisfying DN explanation of the dissolution of salt to append adjunct claims about the dissolution of “hexed salt” (see Kyburg ; Salmon 1989). In doing so, we included irrelevant information, since the hexing presumably plays no part in the dissolving of salt. However, we do not defeat a DN explanation by conjoining it with adjunct, irrelevant premises—though perhaps we worsen it markedly. Note, if we had changed the explanans to say only that “all and only hexed salt dissolves” and removed claims that “all salt dissolves,” then we *would* have failed to provide a DN explanation—since that assumption is inconsistent with our best background theories and evidence. That sort of change is not an addition of complexity, it is a substitution of logically distinct premises. Moreover, the complexity introduced in highly accurate ANNs is not irrelevant in this way; there is no worry that explainability is defeated by magical or irrelevant suppositions about complex MAIS.

The situation is somewhat different for IS explanations. Suppose we have an IS explanation of some phenomenon starting with statistical laws **S** and empirical facts **E** and ending with some inductively inferred conclusion as its explanandum, which might be some particular outcome *x* or a probability of such an outcome *Pr*(*x*) = *p* (see Woodward 1989, 2019). Now assume we discover that some other statistical laws or empirical facts **C** are relevant to the phenomenon in question. Notice that additional statistical laws or empirical facts will, under certain conditions, affect (the probability of) the explanandum. However, our aim is not to show that increasing the complexity of the explanans does not affect the support for the explanandum. That is, if *x* follows inductively from **S** + **E** with support *p* (or *Pr*(*x*) = *p* follows inductively from S + E), then *x* follows inductively from **S** + **E** + **C** with support *p* ± *c* (or *Pr*(*x*) = *p* ± *c* follows inductively from **S** + **E** + **C**).^5^ What’s important here is that despite increased complexity, the process of explanation is the same, so the relationship between the explanans and explanandum remains one we are happy to call explanation. Of course, the explanandum may thereafter be explained with low probability, making the explanation less “successful” or “good,” but not making the explanandum less explainable by the inductive process of IS explanation.

Similarly, increasing the complexity of the explanans in CM explanations does not make the explanandum any less explainable. Consider a CM explanation which appeals to a particular set of causal processes **P** and set of causal interactions **I** which together bring about phenomenon *x*. We might discover additional causal processes and/or causal interactions, which are relevant to *x* (say by uncovering finer grained details about the particular causal process) such that a more complex set of causal processes P* and/or set of causal interactions I* together bring about *x*. While these new sets of causal features may make the explanation harder to follow, they do not make the CM explanation any less an explanation than before. In other words, just because P* + I* is more complex than **P** + **I**, does not make *x* any less explainable by the former than it is by the latter. Similarly for an NM explanation. Such an explanation will depict relevant entities **E** and activities **A** as responsible for the phenomenon *x* in question. While the discovery of additional entities E* and/or activities A* may make the explanation of *x* less intelligible, it does not make the phenomenon any less explainable than before. Complex mechanisms are mechanisms no less.

Indeed, there may be system dynamics of some phenomena, such as a high degree of stochasticity (Skillings 2015; Godfrey-Smith 2016), that make the discovery and articulation of mechanisms or causal processes difficult, or proposed mechanisms dissatisfying. Nonetheless, granted some existing mechanism or process, adding complexity to it in the form of entities and activities, even stochastic activities, does not destroy the mechanism. Similarly, some may worry that complex ANNs are nondecomposable, which is to say, they are impossible to (mechanistically) explain because they are so hierarchically complex that we cannot assume that the interactions between the relevant entities and their activities can be described linearly (see Bechtel and Richardson 2010). However, even if concerns surrounding nondecomposability are warranted in other domains, they do not apply to ANNs since such systems, as we argue below, can be described in NM terms.

Together, we take these considerations to establish the indefeasibility thesis as stated above. The reader may justly worry that this thesis has been bought at too hefty a price, since they are asked to admit many “bad” or “dissatisfying” cases as explanations. In part, this is just the price of doing science, where many explanations, perhaps even many we find good or satisfying today, turn out not to live up to the standards of good explanation. Of particular interest in this connection is when an explanation is deemed bad or dissatisfying because it does not produce understanding. Happily, some of the procedures of science in general and ML in particular aim directly at remedying this by producing better or more satisfying explanations from those less so. We define and explicate these procedures in the context of ML below (Sections 4.1–4.4), collectively under the heading of interpretability methods. Some of these bad explanations are remedied in the course of good science. For now, we turn to the specifics of applying these varieties of explanation to MAIS, confident that the complexity added to MAIS is immaterial to explainability, even if dissatisfying.

## The Explanation in the Machine: Medical AI Systems

Having established the indefeasibility of explanation, we can apply it to the explainability of MAIS. In this section, we argue that even extremely complex MAIS can be explained using each of the models of explanation outlined above. The upshot here is that the trade-off problem is not one of explainability—we do have access to explanations of MAIS. Taken together, each account of explanation applies to ANNs as it would to any other scientific phenomenon of interest, what differs is our capacity to *understand* these explanations. The precise problem is the difficultly of understanding the explanations of complex ANNs. This is because, in contrast to explanation, understanding is defeasible. The AE trade-off problem should therefore strictly be cast as an issue of providing *understanding* of complex AI systems. Conveniently, a plausible solution to this problem has already been proposed: interpretability. A further problem, however, is that there is little agreement over exactly what interpretability is, an issue we deal with in Section 4.

### Medical AI Systems Are Explainable

It is possible to explain MAIS using each of the four models outlined above. What follows are explanation-sketches (see Hempel 1965) of how ANNs can be explained using each model. To illustrate, take a recent deep learning MAIS, developed by MIT and MGH researchers, which is designed to identify and assess dense breast tissue, an independent risk factor for breast cancer. An associated study shows that this system closely matches the reliability of expert radiologists in evaluating breast tissue density and that the MAIS has been successfully deployed in a clinic setting (Lehman et al. 2019). It is built on a ResNet-18 convolutional neural network (CNN) used for computer vision tasks.^6^ The model was trained and tested using a dataset of 58,894 randomly selected mammograms from 39,272 women between January 2009 and May 2011. Of these, 41,479 were used for training and 8,977 for testing. In the testing phase, the MAIS matched with expert assessment at 77% across a standard Breast Imaging Reporting and Data System (BI-RADS), which categorizes tissue in four ways: fatty, scattered, heterogenous, and dense. In binary tests, either dense or non-dense tissue, the model matched expert assessments at 87%. Subsequently, the MAIS was deployed for testing in a clinical setting at MGH and performed well, matching with expert assessments across all four BI-RADS categories at 90% and at 94% for binary tests. The basic idea is that the MAIS takes inputted images and outputs a classification according to categories for breast cancer risk.

To understand how this MAIS can be explained, we must know a little about its system architecture. The ResNet-18 consists of 18 layers. The first 17 layers are all convolution layers, and the last is a fully connected network. This type of ANN model is most commonly used to classify images—it takes an image and outputs a classification.^7^ This is done by feeding the image to the ANN’s input layer as a set of numbers which then get processed through the ANNs weighted nodes and edges and outputted as a vector of real numbers. Typically, the image will then be assigned to the most probable class.

The DN model can be used to explain any particular image classification produced by this MAIS. A DN explanation of how the MAIS assesses an input image involves listing the weights attached to each and every node and the informational routes indicated by each and every edge at every convolution stage, and the weights of the fully connected network along with the assigned numerical values being fed into the input layer and the network architecture. Once we have that, we can list the values for the classifications the MAIS learned in the training and testing phases of development, and see that its classification of the image is based on comparing the ranges of these classifications with the output value of the image. In doing so, we are explaining the explanandum—here, the MAIS classifying of image *I* as classification *c*—using an explanans consisting of a law-like premises—in this case, how the weights of all relevant nodes and edges produced the output value, along with the law that an output is assigned to the most probable class—and additional information about *I*—which includes the set of input values assigned to *I*, and the output value *c*.

While the DN model gives us an explanation of specific classifications, the IS model can help explain the probability that the MAIS’s classifications are accurate. Broadly, the accuracy of the outputs of ANNs can be explained by appealing to details about the training process as statistical laws and the nature of the training data used as empirical information.^8^ If these, taken together, inductively entail a probability that the ANN’s outputs are accurate, then we have successfully explained the accuracy of the ANN’s outputs. In the case of the MAIS above, we can explain its high degree of accuracy, here the matching with expert assessments, by citing the training procedure and details about the mammogram image dataset used.

For CM explanations of ANNs, we would cite the causal processes and causal interactions involved. This would entail describing the ANN using oft-used terms of art, drawing on biological analogies. ANNs are constituted by connected *nodes*, sometimes referred to as artificial neurons that, like biological neurons, receive and send information, or *signals*, via connections resembling biological synapses, termed *edges*. Signals are typically approximations of real numbers that, when received by nodes as *inputs*, are processed using some non-linear function of their weighted sum, and sent as *outputs* to other nodes. Nodes typically have weights (and biases), which are tuned when the ANN is trained. These weights control the strength of output signals and can, in the case of nodes with thresholds, determine whether a signal is sent at all. The nodes in ANNs are typically organized into *layers*, which can perform different tasks by processing their inputs in different ways. Two layers are special: the *input layer* where information is fed into the ANN and the *output layer* which returns a result. Typically, each of the output nodes provides a probability that the input belongs to a particular class. The connections between nodes are essentially functions of the data that is fed into them. Information is fed into the input layer and passes through the different layers until it reaches the output layer, producing a classification. This sort of description can be applied to particular ANNs like the MAIS above. Feeding the mammogram image into the input layer of the ResNet-18 and the convolutional operations of each layer are causal processes, while the signals sent between the different nodes and layers are causal interactions. These causal processes and interactions lead to the output of the MAIS.

For NM explanations of ANNs one has a choice between levels. We can explain the phenomenon of classification at a high level of abstraction, including in our mechanism only the coarse input-output depiction of an ANN; at the level of the abstraction of nodes, signals, and edges; or delve into the details of a mechanistic explanation at the level of the hardware circuitry involved in computation. Jacobson (1959), for example, shows how circuit-board diagrams generally can be abstracted into simpler networks of nodes and connecting edges, and the same process can, in principle, apply to any ANN. A circuit or hardware-level mechanistic depiction requires specifying the relevant entities and activities involved and depicting how their arrangement and interconnection results in the computation of a classification output from inputted image data. Arguably though, this sort of reduction to electrical signals and processes of computation is not the ideal level of mechanistic explanation. Machamer et al. describe the importance of selecting the right level of explanation in terms of “bottoming out” of mechanisms—describing mechanisms typically ends at a level “accepted as relatively fundamental or taken as unproblematic” (2000: 13) by a given discipline. In the context of explaining MAIS classification, we can presumably treat hardware processes as elementary and focus on ANN architecture. At least, until there is some indication that features of MAIS classification turn on lower level mechanisms, the explanation ends there.

Just as with CM explanation above, the essential features of an NM explanation relevant to MAIS will involve identifying entities (nodes and layers) and activities (signaling, input and output), start-up conditions (input data and configuration of the network) and termination conditions (output of and thresholds for classification), the essentials of which are described above. Indeed, there may also be “higher level” mechanistic explanations, which identify more intuitive activities of specific layers or nodes, and these might be more easily “understood” or “visualized” than the mechanistic depiction of the architecture of ANNs (see Section 4.1, and Lipton (2018) on decomposability). But the existence of higher-level explanations does not prevent NM explanation of MAIS at the level of ANNs themselves.

We stress the importance of distinguishing between whether a mechanism is satisfactory or good at some level of abstraction, and whether it is a genuine NM explanation. There is justifiable concern about whether a given NM explanation of an ANN is a good one, particularly when that ANN itself is treated as a model of some other phenomena we are interested in explaining (e.g., the mammalian neocortex, see Buckner 2019). But the provision of an NM explanation must of course precede assessment of its quality. Moreover, MAIS are in some ways actually simpler than the usual targets for NM explanations—biological neurons—since the NM explanations of MAIS in their current form need not account for chemical or analogue features of artificial neurons.

The above discussion illustrates that explanations of ANNs are available to us. The AE trade-off, posed as an issue of “explainability” as it commonly is in ML literature, is therefore not as problematic as one might think.

It is worth considering a possible challenge to our position. Some have argued that the traditional accounts of explanation outlined above are incomplete because they do not take pragmatic elements into account (e.g., van Fraassen 1980; Achinstein 1983). For instance, it has been suggested that explanations are only *good* or *successful* when they provide understanding, and that explanations should exhibit a proper relationship to their audience (Potochnik 2016). Consequently, some may contend that our view fails to adequately account for the context or subject for which a given explanation is intended. According to this objection, the explanations of ANNs provided by the traditional models outlined above should not really be considered explanations at all since they do not guarantee understanding of the outputs given to us by AI systems. While our position is bound to be contentious to some ML researchers and philosophers favoring pragmatic accounts of explanation, there are several reasons for preferring it over such accounts—particularly in efforts to disentangle interpretability from explainability.

First, our view is compatible with widely accepted pluralism about explanation. Pragmatic accounts are also pluralist, but part of the pragmatist argument against traditional accounts is that these assume some overarching account of explanation which covers all contexts, but this is misguided. When explaining ANNs, we can use the account most suited to our given aims. That is, when providing explanations of these traditional sorts, we can account for features of explanatory contexts without adopting a pragmatic account of explanation itself. Second, these traditional models of explanation share a common structure (Fig. 1) that we find helpful in defining interpretation. Indeed, since pragmatists do not dispute this common structure but add to it, the general features of our account of interpretation are adaptable to pragmatic accounts of explanation. Only, they will need to employ more conceptual machinery than necessary to provide an analogous account of interpretability.

Third, and most important, one of the aims of this paper is to argue for the value of separating explanation and understanding in the context of XAI. That is not to say that these concepts are wholly separate. In the next subsection, we contend, in line with much recent work in philosophy of understanding, that explanation and understanding are indeed related, just not as strictly as many ML researchers and proponents of pragmatic accounts think. Further to this, by setting these notions apart we demonstrate that the problem of complexity really lies in its tendency to trade off against understandability. This is crucial to developing an account of interpretability which successfully describes many of the methods used by many ML researchers for increasing understanding of ANNs.

### Separating Explanation and Understanding

Before developing an account of interpretability we must parse part of the relationship between explanation and understanding. There has recently been a surge of philosophical interest in the concept of understanding (see de Regt et al.^2009^; Strevens 2011, 2013; Potochnik 2016; de Regt 2017; Khalifa 2017). Although a full characterization of the concept is well beyond the scope of this paper, these existing accounts illuminate the important differences between understanding and explanation which, we argue, illustrate the defeasibility of understanding.

Some contemporary accounts of science affect somewhat of a revolution by switching focus from explanation to understanding. Because of this, some may be tempted to align them with the pragmatic notions of explanation referred to previously. Common to these accounts, however, are the claims that (1) understanding necessarily involves *having an explanation* and (2) understanding demands satisfying some other condition(s) which are *not dependent on the qualities of the explanation alone*.^9^ Though, there is little consensus about what these other conditions are. Most agree with Potochnik (2016) that this will involve some relationship between the explanation and the explainer or audience, but disagree about what relationship is required. For instance, de Regt holds that, “A phenomenon P is understood scientifically if and only if *there is an explanation* of P that is based on *an intelligible theory* T and conforms to the *basic epistemic values* of empirical adequacy and internal consistency” (2017, p. 93, our emphasis). Strevens (2013) argues that understanding involves “grasping” *a correct scientific explanation*, and characterizes this in terms of possessing a particular *psychological state*. Khalifa (2017) agrees that one understands a phenomenon to the extent that they “grasp” an “explanatory nexus,” but elaborates that grasping refers to a *cognitive state* resembling scientific knowledge.

In stating that an explanation is necessary for understanding, condition (1) helps illustrate the untenability of the claim that if something fails to give rise to understanding, then it is not an explanation. Accepting, in line with much current work on understanding, that (1) is true amounts to accepting that if you understand some phenomenon then you can explain it, or contrapositively that if you cannot explain some phenomenon then you do not understand it. It would be false to conclude from (1) that if you do not understand some phenomenon then you cannot explain it. Simply put, you can explain things you cannot understand—doing just this is surely a part of the learning process and perhaps a psychological preliminary to understanding generally—you just cannot understand things you cannot explain.

Where explanation and understanding are set apart is in terms of (2). Whatever particular view of understanding one may prefer—whether it’s “grasping,” “intelligibility,” “apprehending,” or “knowing”—it is, at least in part, subjective or contextual. The intelligibility of a scientific theory, which is necessary for understanding in de Regt’s account, is by his own lights dependent on a scientist’s being able to “recognize the qualitatively characteristic consequences of T without performing exact calculations” (2017, p. 102; also see de Regt and Dieks 2005). What this means is that intelligibility, and thus understanding, partially relies on subjective features of the individual who is trying to understand the phenomenon in question. For both Khalifa and Strevens, grasping an explanation, and thus whether a given explanation actually provides understanding, will turn on psychological features specific to the user of that explanation, e.g., on features of the scientist, engineer, doctor or patient. Plainly, what is understandable to Sally might not be to John; what is understandable to the engineer of an ANN may not be to a radiologist or the person using a MAIS.

Taken together, (1) and (2) show two things: First, the pragmatic objection to the explainability of ANNs is misguided, thus strengthening the argument that explainability is guaranteed by the sorts of things that appear in the explanans and their relationship to the explanandum. That is why explainability is indefeasible by increasing complexity: The properties of the explanans and relationship to the explanandum remain the same come whatever complexity.

Second, because one’s understanding is conditional on subjective features (whether psychological, cognitive, or contextual), it is not impervious to complexity. The ability some agent has to understand varies between individuals and is sensitive to the complexity of both the explanans and the process of relating it to some explanandum, phenomenon. Understanding, in contrast to explanation, *is* defeasible. If ANNs are strictly speaking explainable but often those explanations are not understandable, then what is needed is methods for making those explanations more understandable. This is precisely the reason that we need to move beyond mere individual explanations to interpretation.

## Interpretability

[T]he paucity of critical writing in the machine learning community is problematic. When we have a solid problem formulation, flaws in methodology can be addressed by articulating new methods. But when the problem formulation itself is flawed, neither algorithms nor experiments are sufficient to address the underlying problem. (Lipton 2018)

We contend that much confusion in the debate about, and push for, XAI can be attributed to a conflation of explainability and interpretability. Interpretation has been devised and variously defined within the sciences themselves (Ribeiro et al. 2016; Mittelstadt et al. 2019; Lipton 2018; Krishnan 2019; Páez 2019). We cannot fault philosophers or scientists for misunderstanding the scientific notion of interpretation since there is no single such notion to rely on. Indeed, interpretability is often connected directly with explanation and understanding, although by way of equation or equivocation which we find unhelpful. Lipton (2018) says of interpretability that it “reflects several distinct concepts,” which is to say that it is used inconsistently, or at best equivocally. Indeed some, like Miller (2019, 8), are happy to accept the equation of interpretability with explainability. Our view is that is it best to set these concepts apart. We begin below with an account of interpretation, from which an account of interpretability follows straightforwardly as the ability to provide an interpretation.

### What is Interpretation?

Fundamentally, *interpretation is something that one does to an explanation to make it more understandable*. When we do not find an explanation understandable, we ask for it to be interpreted. We can also talk of “interpreting data,” “interpreting a phenomenon,” or “interpreting an ANN” but when these do not mean precisely the same thing as “explaining data,” “explaining a phenomenon,” or “explaining an ANN” then these uses are derivative of the notion of interpretability we propose here. To “interpret data” is to do something to an explanation of/involving data; to interpret an ANN is to do something to an explanation of/involving the ANN. An interpretation is also something that is performed on each essential part of an explanation: one interprets an explanans, process of explanation, and explanandum.^10^ An interpretation then gives rise to *another explanation*, by one or a combination of the techniques of XAI, or interpretability methods detailed below (Sections 4.2–4.4). Finally, when successful, an interpretation gives rise to an explanation that is, in some way or another, *more understandable* than the explanation we began with.

Take the example of the breast tissue classifier from before: We might start with a traditional explanation, such as a DN explanation, of a particular output from the MAIS. Given the complexity of such an explanation, it may be extremely difficult to understand for most users; thus, one may ask for a more understandable explanation. We may then apply one of the interpretability methods with the aim of producing a new, more understandable explanation. To generate such understanding, one might restrict an explanation to only that information pertaining to how an individual patient was classified (local interpretation Section 4.3 below). In doing so, we obtain a new explanation from the old, although one presumably less complex—since localized to a single case—and hopefully thereby more understandable.

Terminology about interpretation methods often differs without changing the overall concept. Zednik (2019), for example, identifies a number of techniques to “render opaque computing systems transparent” (p. 8), such as input heatmapping and feature-detector visualization. To our minds, insofar as transparency involves production of explanations that are more understandable, these are cases of interpretation (specifically local and approximate interpretation Sections 4.3–4.4). Watson and Floridi (2020) offer a formal framework, which they refer to as an “explanation game,” by which proposed explanations are scored on three properties: Accuracy, simplicity, and relevance. Accuracy refers to how precisely the explanation models the ANN’s processes from input to output. Simplicity is equated with the understandability of the explanation for a given stakeholder. And relevance refers to whether the elements cited in the explanation are indeed responsible for the output being explained. On this view, the goal of interpretable machine learning is to provide explanations that maximize the balance between accuracy, simplicity, and relevance. Given that the explanation game aims at producing explanations that are more understandable for a given user, we take this framework to be describing cases of interpretation (specifically total and approximate interpretation Sections 4.2 and 4.4). Indeed, that there is something like a connection to the production of greater understanding is perhaps the only consistent feature across accounts of interpretability on offer.

Lipton (2018) and Krishnan (2019) make it clear that the notion of interpretability is often undefined or presumed intuitive and implicit by scientists and engineers. Our choice to characterize interpretation at the level of explanations themselves is in part an attempt to remedy this by providing a workable definition of the *process* of interpretation. Furthermore, this view of interpretation does capture some of the popular formulations of the notion offered within the ML community. Our account of interpretation attempts to strike a balance between consistency with, and unification of, scientific usage.^11^

Consider the view of Ribeiro et al. (2016: 1136) on explanation of ANNs: “An essential criterion for explanations is that they must be **interpretable**, i.e., provide qualitative understanding between the input variables and the response.” One way to read this is as asserting that interpretability is merely synonymous with understandability; another is to see it as a special case. If an explanation is already understandable, then surely it is interpretable, since it gives rise to an understandable explanation (itself), trivially. But, as we have noted, not all interpretable explanations themselves provide understanding—qualitative or otherwise. For that, we may have to wait for an explanation to be interpreted and to give rise to another explanation capable of providing the understanding lacking from the first.

**Fig. 4:**
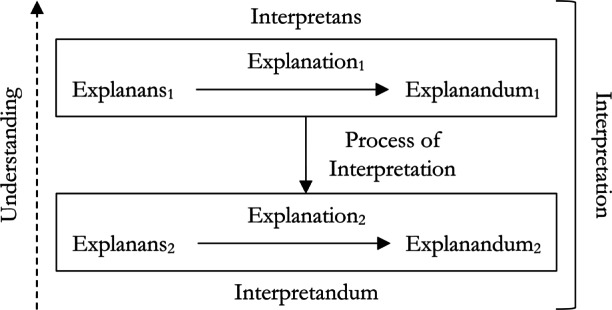
The general structure of total interpretation

We find it helpful to put this view of interpretation in vernacular like that used to discuss the general structure of explanation (Fig. 1). While in explanation the explanandum is some phenomena, diagram, or sentence asserting or describing a phenomenon, in interpretation the thing being interpreted, the *interpretandum*, is an explanation we begin with and that we find difficult to understand. Likewise, while in explanation the explanans is a set of propositions which together explain the explanandum, in an interpretation, the *interpretans* also is an explanation (perhaps conjoined with some additional premises or observations) provided with the intention of being more easily understood. Overall, to provide an interpretation is to show how the interpretans relates to the interpretandum via the *process of interpretation* (Fig. 4).

Given this general structure of interpretation, we can then begin to classify interpretation strategies and methods. In the remainder of this section we offer a preliminary classification of interpretation methods with special attention given to their application within MAIS. For instance, the interpretans might be shown by the process of interpretation to be an *approximation* of the interpretandum, it might be *isomorphic* to (but more familiar than) the interpretandum, or it might match the interpretandum only *locally*, within a narrower range of explananda we find more easily understood. These cases are presented in detail below.

### Total and Partial Interpretation

As noted above, both the interpretans and the interpretandum are explanations; each consists of an explanans, explanation process, and explanandum. A case of *total* interpretation is one in which the interpretans is totally different from the interpretandum. In other words, one in which the explanans, explanation process, and explanandum contained in the former differ in some way from the explanans, explanation process, and explanandum of the latter. For example, consider a simple substitution of variables. Suppose we have an explanation, derived from an ANN in a medical context, that mentions some variable *v* in each essential part, the explanandum itself being some claim about *v*, e.g., that it is relevant to breast tissue classification. Coming to this explanation without background information about the meaning of *v*, we might likewise not understand the meaning of the explanandum. But supposing we are allowed to provide such a meaning by further investigating the set-up of the MAIS, e.g., *v* = *radiologically white tissue*, we can construct a new explanation that specifies this meaning in place of *v* in every essential part, thus changing every part of the explanation. Here, the replacement of “*v*” by “*radiologically white tissue*” in every part of an explanation, generating a new explanation without “*v*,” is a process of total interpretation. The new explanation, for example, will not explain that “*v*” is relevant to breast tissue classification, but that the presence of radiologically white tissue is relevant to breast tissue classification.^12^

**Fig. 5:**
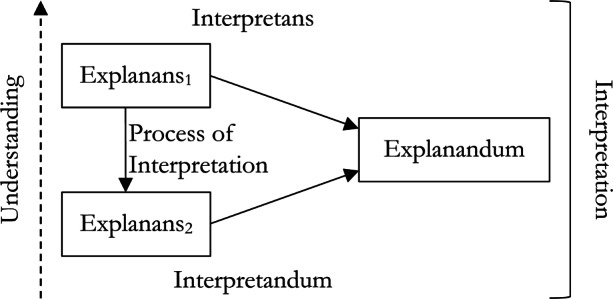
A partial interpretation wherein the explanandum remains the same in both the interpretans and interpretandum

Often, when we fail to understand an explanation of some phenomenon *x*, we want to interpret this explanation to provide understanding, but still want to obtain an *explanation of x*. In such cases, what we want is to “adduce” an interpretans, the explanandum of which is identical to the explanandum of the interpretandum (diagrammed in the triangular Fig. 5, a special case of Fig. 4). That is, we can sometimes provide a *partial* interpretation by showing how one explanans arises from another, by some process of interpretation, itself providing some explanation of the very same explanandum. Put another way, a partial interpretation is just a re-explanation of the same explanandum, equipped with a relationship between the new explanans and the old.

**Fig. 6:**
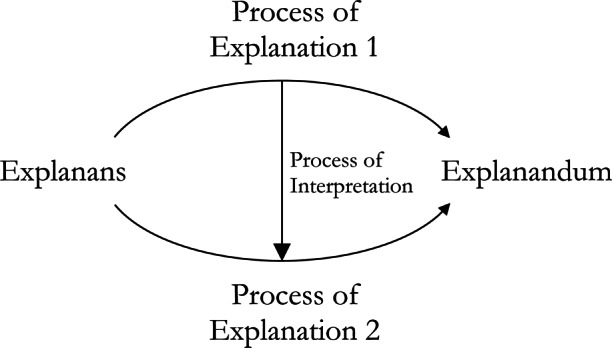
A partial interpretation wherein the process of interpretation used results in a new process of explanation for an explanans and explanandum

This sort of interpretation is often what is aimed for early on in efforts to generate understandable explanations, since the desiderata of the initial explanation of *x* is understanding of *x*, and remains so after the demand for interpretation. Indeed, another sort of partial interpretation involves interpreting only the process of explanation itself. Supposing we are given a DN explanation, where the process of explanation, the *deduction*, is too complicated or long to be held in mind—consider any explanation which requires a mathematical proof that relies on outsourcing steps to a computer, such as Gonthier’s (2005) proof of the four color problem. Here the aim is not to adduce any new explanans or explanandum, these are fixed, but to find some new process of explanation, e.g., a more simple or succinct deduction. This case is diagrammed in Fig. 6.

Failing a partial interpretation, we may aim to provide a total interpretation. But this immediately raises a problem: what should the relationship between two explanations be such that I come to better understand one by coming to understand the other? If I aim to understand *x* but its explanation *E* is unintelligible to me, and I am given a total interpretation such that a second explanation $E^{\prime }$ explains $x^{\prime }$, then there ought to be a special relationship between *E* and $E^{\prime }$ if the latter is to provide any understanding whatever about *x*.

Another way to put the problem of relating different explananda in a total interpretation is by asking what sorts of relationships between phenomena are relevant to scientific explanation: Similar phenomena can sometimes figure in explanations with the same overall structure. For example, explanations of tissue classifications based on radiological whiteness can inform explanations of classifications based on tissue density. Provided we have some justification for substituting radiological whiteness for tissue density, explanations on the basis of radiological whiteness can be totally interpreted by those referring to tissue density instead. The hope being that tissue density provides some understanding that radiological whiteness does not. Of course, the similarity of two phenomena does not *imply* that there should be any understanding gained about one via explanation of the other—understanding is too psychologically contingent for this—but such relationships are, methodologically, good places to begin the search for understanding.

In the context of formal explanations, where our concern is not phenomena themselves but the diagrams or descriptions thereof, these observations manifest as strategies or methodologies of interpretation. That is, we can further classify total interpretations according to whether the interpretans and interpretandum are related by *approximation* or by *isomorphism*—e.g., if *Eapproximates* $E^{\prime }$, then we have some reason to think that $E^{\prime }$ *can provide understanding about x*. These two cases are detailed below, but since both of these methods come in “local” and “global” forms, we address these notions first.

### Local and Global Interpretation

The ML literature is replete with claims about “local” explanation and interpretability (see Ribeiro et al. 2016, 2018; Doshi-Velez and Kim^2017^; Adebayo et al. 2018) that, given the accounts of explanation favored among philosophers, are liable to confuse. This is because the accounts of explanation are classified according to the nature of their explanans (laws of nature/statistical laws/entities and activities) and the process of explanation (deduction/induction/depiction), while the designation of an interpretation as “local” is a comparative property of explananda. To our minds the distinction between local and global interpretations is best captured as follows. Considering a pair of explanations *E* and $E^{\prime }$, with respective explananda *D* and $ D^{\prime } $, $ E^{\prime } $ is local relative to E if and only if $ D^{\prime } $ is a subset of *D*. There are many methods of doing this current in ML, however, common to all is the identification of such a subset.

Consider for a common example the Universal Law of Gravitation (ULG), which states that the force of gravitation between any two objects is equal to the product of their masses over the square of the distance between them times the gravitational constant. We can use this law in conjunction with a set of observations (*O*) about the masses and distances separating each planet in our solar system to explain (or predict) the force that would (absent other interactions) attract every pair of planets (*G*). Take this to be an explanation $E: ULG + O \rightarrow G$. If we restrict ourselves to explaining only the forces between each planet and earth (*L*), we may likewise restrict the *ULG* to the following Earthly Law of Gravitation (ELG): the forces of gravitation between Earth and any other body is equal to the gravitational constant on earth times the mass of the body over the square of the distance of that body from earth. Note that inclusion of explananda can arise due to implication between explanans’. In this case, ULG implies ELG but not vice versa, and this gives rise to an explanation $E^{\prime }: ELG + O \rightarrow L$. Finally, since *L* is a subset of *G*, $E^{\prime }$ is local relative to *E*.

In the context of ML, a function-based example is also illuminating. Consider the ANN which has learned to output the absolute value of its inputs. A (DN) explanation of the output of this ANN will consist of the statement of the functional “law” together with some input “observations,” these being the explanans, concluding with the statement of the output as explanandum. This can essentially be seen as the function *h*(*x*) = |*x*|, giving the explanation *E* : *“h*(*x*) = |*x*|” + *“O* = {*x*_*i*_ = − 1,*x*_*i*+ 1_ = 1,...}” $\rightarrow ``D=\{x_{i}=|-1|, x_{i+1}=|1|,...\}$.” Another explanation that only explains the outputs of *positive* inputs could likewise restrict the function *h*(*x*) to *x* > 0, which is essentially the same as the function *f*(*x*) = *x*, giving the explanation $E^{\prime } : ``f(x) = x$” + *“O* = {*x*_*i*_ = − 1,*x*_*i*+ 1_ = 1,...}” $\rightarrow ``D^{\prime }=\{x_{i+1}= 1,...\}$.” Since $D^{\prime }$ is a subset of *D*, we conclude that $E^{\prime }$ is local relative to *E*. Here, to “adduce” a local explanation involved recognizing that *h*(*x*)|_*x*> 0_ = *f*(*x*)|_*x*> 0_ = *x*, and while in practice applications of constructing local explanations are surely more complex than this, they each share this general theme: once we have decided to explain a subset of our explanandum, we can sometimes make this work by adducing a restriction on our explanans—one intended to make the new explanation more understandable.

Thus far we have just described “locality” as a comparative property of explanations and based entirely on the inclusion of explananda. In practice, we usually start with one complex explanation, and wish to interpret it, i.e., provide another, more understandable explanation. One way to achieve this is to localize the explanation by adducing another explanation that is local with respect to the explanation we began with. Many methods in ML (e.g., LIME, SHAP) identify this local subset of *D* by defining a geometric measure of distance between input values and building a model (e.g., a sparse linear model or shallow tree, Section 4.4) that approximates the more global model in the environment of a particular input, as defined by this distance metric. Once we have an explanation in hand (the interpretans) given by the locally trained model, we can check to see that indeed it is local (by the process of interpretation) relative to our starting explanation (the interpretandum). The process of interpretation in such cases is showing that the explanans of the interpretans is somehow restricted from the explanans of the interpretandum, and demonstrating that the explanandum of the interpretans is included in that of the interpretandum.

In the case of ANNs, our adduction of a restriction on the explanans amounts to a restriction on the inputs to that ANN in essentially the same manner as described for ordinary functional restrictions. In the most extreme cases of local interpretation we move to an explanation of a single data point, a single classification. And in that somewhat trivializing case, an explanation of the classification of the input can be reduced to a selection of features of the input that most affect the output; such local interpretations provide counterfactual information about which features of the input, if intervened upon, would change the output. For example, local interpretation methods in MAIS often identify the particular pixels, present in an input image, that most affect the classification output (Páez 2019; Zednik 2019; see also Ribeiro et al. 2016). In practice, methods for interpretation of predictions of machine learning algorithms often approximate the explanation, in addition to localizing it to a given datum, as examined in the following section.

### Interpretation by Approximation or Isomorphism

When an explanation is too complex to be understandable to its user, a possible solution is to interpret the explanation by providing another, more understandable and similar explanation. In general, measuring similarity depends on a prior choice of some features to compare—mice may be similar to humans with respect to the efficacy of some medication, but not similar in eloquence. In the context of MAIS, an ANN can be thought of as a highly non-linear function *f*(*x*). Approximating the ANN predictions would entail providing a (more understandable^13^) function *g*(*x*), such that the outputs of *f*(*x*) and *g*(*x*) are similar on some metric, e.g., the square deviation, ${\int \limits }_{X}{(f(x)\ -\ g(x))^{2\ }}$. When an ANN appears in some explanation, this approximation can successfully figure in the process of interpretation precisely when we expect *g*(*x*) to be *more understandable* than *f*(*x*).

Many people find linear functions and decision trees more understandable than ANNs. Indeed, methods for interpretation of ANNs often aim to provide *g*(*x*) that is either linear or tree-like, despite what some see as scant justification that these models really are any more understandable (Lipton 2018). Still, some studies illustrate that sparse rule lists and linear models fare well for human-interpretability (Lage et al. 2019; Narayanan et al. 2018). *Distillation* methods work by training one model to approximate the predictions given by another, and can be done either globally or locally.

Linear models are used to provide *local* explanations. While linear models are not flexible enough to globally approximate a neural network, they can provide adequate accuracy when the explanandum is small, such as a single prediction. For this purpose, various methods have been developed that attempt to determine the set of features that are important to explaining the output of a given prediction (Ribeiro et al. 2016; Mishra et al. 2017; Lundberg and Lee 2017; Chen et al. 2018, 2018). These methods essentially localize the explanation of an ANN prediction to a small neighborhood around a specific prediction (this being the new explanandum) and approximate this localized explanation. For example, for the ANN described above for the prediction of dense breast tissue, while a global explanation of what makes tissue dense might be too complex to understand, an explanation of approximately which pixels in a given mammogram led to it being classified as dense may be simple enough to be understood by visual inspection.

Since linear models are often not flexible enough to provide sufficient global approximations, global ANN distillation in particular involves using decision trees as the approximating model. When trees are more understandable, distillations are thereby a method of interpretation by approximation. Trees and ANNs both have quite a strong property *as approximators*.^14^ Both are “universal approximators,” which means that they can approximate any continuous function to an arbitrary degree of accuracy (Hornik et al. 1989). Since ANNs are continuous functions, decision trees can therefore approximate neural networks to any accuracy.

The process of distilling a neural network into a tree involves generating input data and using ANNs to get a prediction, then training a decision tree on the sets of input-output pairs given by the ANN. One can always generate as much data as needed to achieve a desired level of similarity between the model outputs. Nonetheless, despite the *prima facie* understandability of *small* trees as compared to networks, in practice the distillation of useful ANNs tends to result in *very large* trees (Frosst and Hinton 2017).

Here we are faced with the understandability-accuracy trade-off, since one can also sacrifice accuracy of a tree distillation by reducing or fixing its size or depth (Zhou et al. 2018), thereby presumably purchasing some understandability at the expense of accuracy. Though we may gain some traction of understanding by initially approximating an ANN, the upshot of this is that, as we increase the accuracy of the approximating model, we wash out any understanding gained by approximation itself and are left only with whatever advantage is offered by modelling (nearly) the same process in a different sort of model. When we interpret some explanans that includes an ANN into another including a tree, which approximates the first to an arbitrary degree, we have, in a way, gone beyond approximation into interpretation by isomorphism.

Since understanding has a psychological component, it has some mathematically not well-behaved properties. For one, understanding is not isomorphism invariant. That is, if I understand *a*, the fact that *a* is isomorphic to *b* does *not* imply that I understand *b*. Intuitively, my understanding of some statement of English does not imply anything about my understanding of equivalent sentences in another language. This is because our understanding can turn on features of the specific presentation of some notion and the background knowledge of the user of the explanation. Nonetheless, the converse of this has been put to use as a method of interpretation. Indeed, if I do not understand *a*, I may still understand some isomorphic *b* (due perhaps to my familiarity with constructions in the language of *b*).

Perhaps the ubiquity of trees and tree-like structures in our everyday experience explains the prevalence of tree distillations in ML; their familiarity evidently leads to the idea that they will be understandable, and that may indeed be a reasonable assumption to make. Nonetheless, there is a very wide range of types of models that can universally approximate, or are genuinely isomorphic to, ANNs. Candidate alternative universal approximators include fuzzy systems (Zadeh 1965; Wang 1992; Kosko 1994; Castro 1995; Yen et al., 1998, cf. Klement et al. 1999); Neural ODEs (Chen et al. 2018, 2018; Zhang et al. 2019; and references therein) and nearest-neighbor methods (Cover and Hart 1967; Stone 1977). When we have reason to believe that any of these alternative models are “more understandable” than ANNs, we thereby have reason to make use of these isomorphic models to provide an interpretans, i.e., an explanation using the alternative model within its explanans, to serve the same role that the ANN served in the interpretandum.

## Conclusion

Conceiving of explanation according to those accounts offered within the philosophy of science disentangles the accuracy-explainability trade-off problem in AI, and in doing so, deflates the apparently paradoxical relationship between trust and accuracy which seems to plague debates in medical AI and the ML literature. *If* it is simply that explainability is required for trust, there is no cause for worry, since highly accurate (and potentially complex) MAIS are just as explainable as simple ones. We resolve this issue by using clearly defined accounts of explanation, and demarcating notions of explanation from understanding and interpretation. Our account of interpretation is explicitly positioned as distinct from accounts of explanation, while making clear the connection that both have to understanding.

While explanation is a well-theorized notion within the theory and philosophy of science, “interpretation” and a corresponding notion of “interpretability” are not (see Lipton 2018). We have attempted to synthesize a notion of scientific “interpretability,” from cases where it was *not* used synonymously with either “explainability” or “understandability.” This was done to provide a theoretical framework that generalizes the methods scientists and ML researchers often use for the purpose of interpretation, and to help remedy this lacuna within the ML literature in particular and the neglect of inter-explanatory relationships in philosophy of explanation broadly speaking. In the context of MAIS, there never really was a problem explaining artificial networks. Rather, the problem has always been understanding the explanations that were available, and our account of interpretation shows why.

We define interpretation as a process taking one explanation to another, more understandable explanation. Understandability is at least partly psychological, depending not only on the phenomenon or its explanation but also on the user of the explanation. Identifying the elements of explanations that make them understandable broadly speaking is an active research topic in the social sciences (Miller 2019), but beyond the scope of a theory of interpretation as such. Just as de Regt (2017) says we need a general theory of understanding that is independent of our specific accounts of explanation, we require a general theory of interpretation that is independent of both. Our framework can thereby only be employed once we have made some theoretical choices about what features of explanations indeed provide understanding, without which the success of an interpretation cannot be assessed.

The ubiquitous presumption that only simple and/or linear models are “understandable” is liable to limit the potential scope of scientific interpretation; the use of non-linear and complex models should not be excluded at the outset. It seems a large part of the stress on the point of explainability in discussions of ANNs and MAIS boils down to an insistence that they be understandable to a non-specific and correspondingly broad audience of clinicians, patients, and possibly the general public. With such a diverse audience of users of explanations, perhaps simplicity is the only proxy for understandability, and persistent demands for “explainable” ANNs are reducible to demands for simple and potentially correspondingly weak MAIS. We can move away from this tendency to simplicity by demanding that ANNs be *interpretable* in the sense defined here. That is, by demanding that we find ways to convert explanations that are not understood into those that are more understandable in a user-relative way. That way, we might keep many complex and thus strong MAIS while achieving broad understandability by, counter-intuitively, adding further “simplifying complexity” in the form of interpretation methods.

## Footnotes

1 Readers familiar with these accounts of explanation may safely skip to Section 2.2.

2 Prior to this, Salmon (1971) proposed his statistical relevance model of explanation. Although we do not cover it here, it is worth noting that he does not wholly abandon the notion of statistical relevance, but rather claims that one typically acquires information about statistically relevant relations before providing a CM explanation.

3 This conception of explanation derives from examination of molecular-biology and neurophysiology, specifically, mechanisms of protein synthesis and neurotransmitter release.

4 As Levy (2013) notes, advocates of the explanatory value of mechanisms are “fairly coy” (p. 102) about defining the key notions, a matter further complicated by a lack of universal terminology. E.g., sometimes “parts” are discussed instead of “entities” (Bechtel 2006). However, for present purposes only the outlines of the NM approach are required.

5 There are cases where the addition of an empirical fact may lead to a deductive explanation, for our purpose we can treat this as a trivial case of inductive explanation, where the probability is zero or one.

6 A CNN is an ANN particularly adept at detecting patterns in images. It uses convolutional layers to split an image up into overlapping tiles, which are then analyzed for learned patterns before the signal is outputted to subsequent layers (see Goodfellow et al. 2016, Chapter 9).

7 While we use an example of a diagnostic MAIS, viz. a classifier, our arguments apply to ANNs designed for other goals, such as automated planning and personalized recommendations.

8 Karimi et al. (2020) deploy an approach that allows assessment of the classifications of ANNs on the basis of causal information associated with the generation of training data. Provided such causal information is obtained by statistical assessment of the target for MAIS classification, it can figure in either IS or CM explanations of ANN accuracy.

9 A notable exception is Lipton (2009), who argues that we can have understanding without explanation. However others, including Strevens (2013) and Khalifa (2017), have disputed this.

10 Although in real cases, many of these components of interpretation may be implicit or trivial, i.e., one may leave the explanandum unaffected (see Section 4.2 below on partial interpretations).

11 Providing an analysis of interpretation also runs into some of the same problems as philosophical accounts of explanation, e.g., whether there is a specifically scientific notion of interpretation that is at issue, or whether the idea is somehow continuous with quotidian examples such as “interpreting a work of art” or “interpreting a foreign language.” That said, at least part of the success of philosophical accounts of scientific explanation have been in their apparent ability to accommodate at least some of the more common prima facie non-scientific cases of explanation, and we believe an account of interpretation can follow suit.

12 Notice that, by replacing variables everywhere in an explanation we also change the process of explanation. Supposing for example that the explanation was DN, the change of variables will change the content of the deduction of the explanandum from the explanans.

13 Approximations of ANNs also have roles besides interpretation, e.g., saving computational time or facilitating analysis, but are here considered only for that role.

14 Importantly, this does not mean they are equivalent at learning tasks.
